# Thermal Conductivity of a Nanoscale Yttrium Iron Garnet Thin-Film Prepared by the Sol-Gel Process

**DOI:** 10.3390/nano7090247

**Published:** 2017-08-31

**Authors:** Yun Young Kim

**Affiliations:** Division of Mechanical, Automotive, and Robot Component Engineering, Dong-eui University, Busan 47340, Korea; ykim@deu.ac.kr; Tel.: +82-51-890-1649

**Keywords:** yttrium iron garnet, thin-film, sol-gel process, thermal conductivity, ultrafast pump-probe technique, materials characterization

## Abstract

The thermal conductivity of a nanoscale yttrium iron garnet (Y_3_Fe_5_O_12_, YIG) thin-film prepared by a sol-gel method was evaluated using the ultrafast pump-probe technique in the present study. The thermoreflectance change on the surface of a 250 nm thick YIG film, induced by the irradiation of femtosecond laser pulses, was measured, and curve fitting of a numerical solution for the transient heat conduction equation to the experimental data was performed using the finite difference method in order to extract the thermal property. Results show that the film’s thermal conductivity is 22–83% higher than the properties of bulk YIG materials prepared by different fabrication techniques, reflecting the microstructural characteristics and quality of the film.

## 1. Introduction

Yttrium iron garnet (Y_3_Fe_5_O_12_, YIG) is a ferrimagnetic material that is promising for applications in microwave communication systems and magneto-optic devices because of its distinctive magnetic and structural properties such as the narrow linewidth of the ferromagnetic resonance, low magnetic loss, and moderate saturation magnetization [1]. Especially with the evolution of microfabrication techniques, novel thin-film based magnetic sensors and data storage media are developed, and consequently the fabrication and characterization of nanoscale YIG films have become important research topics since their properties are often different from those of the bulk form and the device performance is strongly influenced by fabrication techniques that determine the film quality and microstructures [2]. To grow a YIG film, a variety of deposition methods can be employed, and some examples are microwave sintering [3], liquid phase epitaxy (LPE) [4], radio frequency magnetron sputtering [5], pulsed laser deposition (PLD) [6], plasma spraying [7], and the sol-gel method [8]. Among them, the sol-gel method is mainly advantageous in that it is cost-effective [9]. Besides, it can produce films of high purity and homogeneity at lower synthesis temperature [10,11,12,13].

Studies on the magnetic and physical properties of YIG have been actively conducted for more than a decade for applications to microwave oscillators [14], variable delay lines [15], magneto-optical devices [16], and so on. Thermal characteristics, however, still need to be investigated further because the material shows interesting thermodynamic and transport properties due to the magnon-phonon interaction at low temperatures in the presence of a magnetic field [17]. Characteristics at room temperature are also important for the thermal design of devices because it requires actual material properties frequently different from literature values. Therefore, the thermal conductivity of a nanoscale YIG thin-film prepared by the sol-gel method was evaluated in the present study. The ultrafast pump-probe technique was employed for the measurement because it is especially effective for nanoscale materials characterization, providing non-contact and nondestructive ways to measure thermal properties with high temporal and spatial resolutions [18]. It can measure the thermal conductivity and thermal boundary conductance of a very thin film of which thickness is only a few tens of nanometers. Moreover, it can simultaneously evaluate mechanical properties such as the Young’s modulus and longitudinal bulk wave velocity by solving thermoelastically-coupled equations [19,20]. Unlike the 3*ω* method [21], in situ testing is possible because it does not require physical contact for the measurement, and electrically conducting materials can also be measured since it is an optical technique, without the need for patterning metallic strip heaters on the sample surface.

## 2. Materials and Methods

### 2.1. Sample Preparation

A YIG film sample prepared by the sol-gel process in reference [8] was used in this study. A brief description of the sample preparation procedure is as follows: regent grade Yttrium nitrate hexahydrate [Y(NO_3_)_3_·6H_2_O, 99.95% purity] and iron(III) nitrate nanohydrate [Fe(NO_3_)_3_·9H_2_O, ≥98% purity] were dissolved in a 2-methoxyethanol solvent and refluxed at 80 °C for 3 h. The pH was maintained in the range of 2–3 by adding diethylamine to the solution, and it was stirred for 3 days after cooling down to room temperature.

The gel was spin-coated on a quartz substrate at 3500 rpm for 30 s to form a film. It was then baked at 90 °C for 2 h to remove residual solvents and at 350 °C for 15 min to burn off organic compounds. Lastly, the film was annealed at 1000 °C for 2 h at a heating rate of 4 °C/min for crystallization.

Prior to the thermal characterization, a thin layer (70 nm) of aluminum (Al) was deposited on the film surface using an e-beam evaporator (Auto 500, Edwards, West Sussex, UK) for the absorption of laser pulses and measurement of reflectance changes as heat diffuses into the film. The chamber pressure was maintained at 3 × 10^−6^ Torr during the deposition, and a deposition rate of 0.16 nm/s was obtained by applying 4.7 kV DC voltage to a tungsten filament. The film thickness was controlled using a quartz crystal monitor in the chamber. A bare quartz substrate was also installed to prepare a reference sample for the curve fitting to extract the thermal conductivity of Al. The film surface and cross-section were inspected using a focused ion beam system (Scios, FEI, Hillsboro, OR, USA).

### 2.2. Ultrafast Pump-Probe Technique

**Figure 1:**
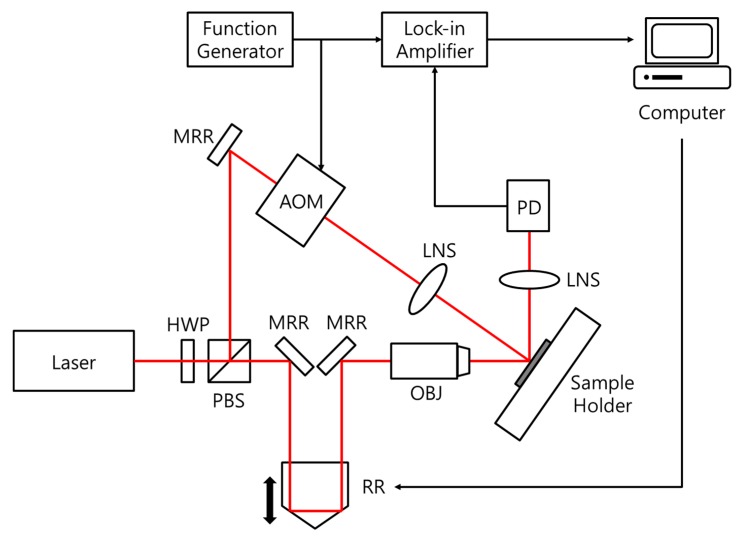
A schematic of the ultrafast pump-probe setup. AOM: acousto-optic modulator; HWP: half-wave plate; LNS: focusing lens; MRR: mirror; OBJ: objective lens; PBS: polarizing beam-splitter; PD: photodetector; RR: retroreflective mirror on a motorized translation stage.

The ultrafast pump-probe setup was used to measure the thermal property of the YIG film. Figure 1 shows a schematic of the setup. A train of femtosecond laser pulses was generated from an ultrafast Ti: Sapphire oscillator (Tsunami, Spectra-Physics, Santa Clara, CA, USA) pumped by a 532 nm wavelength 5 W continuous wave laser (Millenia Pro, Spectra-Physics, Santa Clara, CA, USA). The laser wavelength (*λ*), full width at half maximum (FWHM) pulse width (*τ*), and repetition rate were 780 nm, 120 fs, and 80 MHz, respectively. The beam was divided into the pump and probe paths using a polarizing beam splitter (PBS252, Thorlabs, Newton, NJ, USA) and a half-wave plate (WPH05M-780, Thorlabs, Newton, NJ, USA) at an intensity ratio of 10:1. The pump-beam was modulated at 100 kHz using an acousto-optic modulator (AOM405, IntraAction Corp., Bellwood, IL, USA) and focused on the sample surface while the probe-beam was directed to the heating spot via a retroreflectve mirror on a linear stage (MM-4M-EX, National Aperture Inc., Salem, NH, USA) that can adjust the optical path length and through a 20× microscope objective lens (M Plan APO NIR, Mitutoyo Corporation, Kawasaki, Kanagawa, Japan). The resolution of time delay was optimized to 4 ps/pt since acoustic pulses caused by the propagation of bulk waves in the film are of no interest in this study. The control of the probe delay was automated using LabVIEW (National Instruments, Austin, TX, USA) software during the measurement, and the probe-beam reflected from the sample surface was collected in a photodetector (PDA8A, Thorlabs Inc., Newton, NJ, USA) connected to a lock-in amplifier (SRS830, Stanford Research Systems, Sunnyvale, CA, USA). The lock-in time constant (*τ*_lock-in_) was in the order of ms in order to avoid the effects from previous pump-pulses since the in-phase part of the lock-in response is [22]:

(1) $$V_{\mathrm{LI}}(t)\propto \frac{1}{\delta_{\mathrm{pump}}}\sum_{q=-\infty }^{q=\infty }\cos[2\pi f(q\delta_{\mathrm{pump}}+t_{\mathrm{pp}})]\Delta R(q\delta_{\mathrm{pump}}+t_{\mathrm{pp}})$$

where *V*_LI_ is the in-phase output of the lock-in signal, *δ*_pump_ is the spacing between pump-pulses (12.5 ns), *f* is the pump-pulse modulation frequency (100 kHz), *q* is an integer, *t*_pp_ is the time delay of the probe relative to the pump, and Δ*R*(*t*) is the change in reflectivity that would occur after the application of a single pump-pulse, *t* being the time. Equation (1) is valid when 2π*fδ*_pump_ is much less than unity, and *δ*_pump_ is much less than *τ*_lock-in_. 

## 3. Theory and Calculation

**Figure 2:**
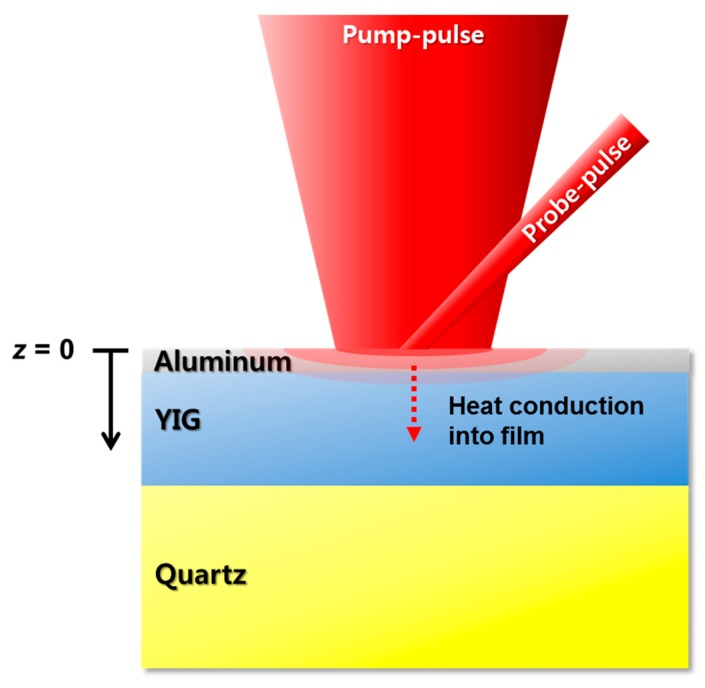
A schematic of the thermoreflectance measurement principle. The pump-pulse heats up the aluminum (Al) sample surface, and the probe-pulse monitors the reflectance change during the heat diffusion into the film thickness direction.

A numerical simulation was performed using the finite different method (FDM), and the thermal conductivity of the YIG sample was evaluated. Figure 2 shows a schematic of the measurement principle. The absorption of the pump-pulse on the film surface results in a sudden increase of reflectance followed by an exponential decay with heat diffusion into the film. Since the reflectance change is proportional to the temperature change, a transient one-dimensional heat conduction equation is calculated and the solution is compared with the measurement data. The governing equation is expressed as follows [19]:

(2) $$\rho C_{p}\frac{\partial T(z,t)}{\partial t}=\kappa \frac{\partial^{2}T(z,t)}{\partial z^{2}}+\dot{g},$$

where *C*_p_ is the specific heat, *T* is the temperature, *z* is the film thickness, *κ* is the thermal conductivity, and *ρ* is the density. The rate of volumetric heat generation, *ġ*, due to the absorption of the laser pulse, is described as follows [19]:

(3) $$\dot{g}=\frac{\beta (1-R)}{2}e^{-\beta z}I(t),$$

where *R* is the optical reflectivity and *β* = 4π*k*_0_/*λ*, *k*_0_ being the extinction coefficient, is the absorption coefficient. Here, *I*(*t*) is the temporal pulse shape [19]:

(4) $$I(t)=\{\begin{array}{cc} I_{0}\sin^{2}\left(\frac{\pi t}{2\tau }\right) & 0<t<2\tau \\ 0 & t<0\;\mathrm{or}\;t>2\tau \end{array},$$

where *I*_0_ is the laser pulse intensity. For boundary conditions, the temperature drop across the Al/YIG and YIG/Quartz interfaces induced by the thermal boundary conductance, *σ*_K_, is considered [23]:

(5) $$-\kappa_{\mathrm{Al}}{\frac{\partial T_{\mathrm{Al}}}{\partial z}|}_{z=d_{1}}=\sigma_{K_{\mathrm{Al}/\mathrm{YIG}}}\left({T_{\mathrm{Al}}|}_{z=d_{1}}-{T_{\mathrm{YIG}}|}_{z=d_{1}}\right),$$

(6) $$-\kappa_{\mathrm{YIG}}{\frac{\partial T_{\mathrm{YIG}}}{\partial z}|}_{z=d_{1}}=\sigma_{K_{\mathrm{Al}/\mathrm{YIG}}}\left({T_{\mathrm{Al}}|}_{z=d_{1}}-{T_{\mathrm{YIG}}|}_{z=d_{1}}\right),$$

(7) $$-\kappa_{\mathrm{YIG}}{\frac{\partial T_{\mathrm{YIG}}}{\partial z}|}_{z=d_{2}}=\sigma_{K_{\mathrm{YIG}/\mathrm{Quartz}}}\left({T_{\mathrm{YIG}}|}_{z=d_{2}}-{T_{\mathrm{Quartz}}|}_{z=d_{2}}\right),$$

(8) $$-\kappa_{\mathrm{Quartz}}{\frac{\partial T_{\mathrm{Quartz}}}{\partial z}|}_{z=d_{2}}=\sigma_{K_{\mathrm{YIG}/\mathrm{Quartz}}}\left({T_{\mathrm{YIG}}|}_{z=d_{2}}-{T_{\mathrm{Quartz}}|}_{z=d_{2}}\right),$$

where *d*_1_ and *d*_2_ are the depths of Al/YIG and YIG/Quartz interfaces from the top surface (*z* = 0), respectively. In addition, the heat flow from Al surface to air is ignored:

(9) $${\frac{\partial T}{\partial z}|}_{z=0}=0,$$

For the initial condition, the film is at rest prior to the application of laser pulses:

(10) $${T|}_{t=0}={\frac{\partial T}{\partial t}|}_{t=0}=0,$$

A Crank–Nicolson approach was adopted for the numerical analysis, resulting in the governing equation in a discretized form as follows:

(11) $$\rho C_{p}\frac{T_{i}^{n+1}-T_{i}^{n}}{\Delta t}=\frac{\kappa }{2}\left[\frac{\left(T_{i+1}^{n+1}-2T_{i}^{n+1}+T_{i-1}^{n+1}\right)+\left(T_{i+1}^{n}-2T_{i}^{n}+T_{i-1}^{n}\right)}{\Delta x^{2}}\right]+\frac{1}{2}\left[{\dot{g}}_{i}^{n+1}+{\dot{g}}_{i}^{n}\right],$$

where *i* is the node number, *n* is the time step, and Δ*x* is the grid spacing.

Once the result is obtained, the normalized surface reflectance change, Δ*R*/*R*, is calculated using the following equation [24]:

(12) $$\frac{\Delta R}{R}=\frac{4\left(n_{0}-k_{0}\right)\left[\left(n_{0}^{2}-k_{0}^{2}-1\right)I_{\eta }-2n_{0}k_{0}J_{\eta }\right]v_{\eta }+8n_{0}k_{0}\left[2n_{0}k_{0}I_{\eta }+\left(n_{0}^{2}-k_{0}^{2}-1\right)J_{\eta }\right]w_{\eta }}{{\left(1+n_{0}^{2}+k_{0}^{2}\right)}^{2}-4n_{0}^{2}},$$

where *n*_0_ is the refractive index, and *v*_η_ and *w*_η_ are the real and imaginary sensitivity coefficients, respectively. *I*_η_ and *J*_η_ are integrals for accounting the depth-dependent contributions [24]:

(13) $$I_{\eta }=K_{0}\int_{0}^{\infty }A_{\eta }(z)\exp\left[-2k_{0}K_{0}z\right]\sin\left(2K_{0}n_{0}z\right)dz,$$

(14) $$J_{\eta }=K_{0}\int_{0}^{\infty }A_{\eta }(z)\exp\left[-2k_{0}K_{0}z\right]\cos\left(2K_{0}n_{0}z\right)dz,$$

where *K*_0_ is the free-space optical wavenumber and *A*_η_(*z*) is the temperature distribution. After calculating the surface reflectance change, the solution is curve fitted to the experimental data so that the thermal conductivity of the sample can be estimated.

## 4. Results and Discussion

**Figure 3:**
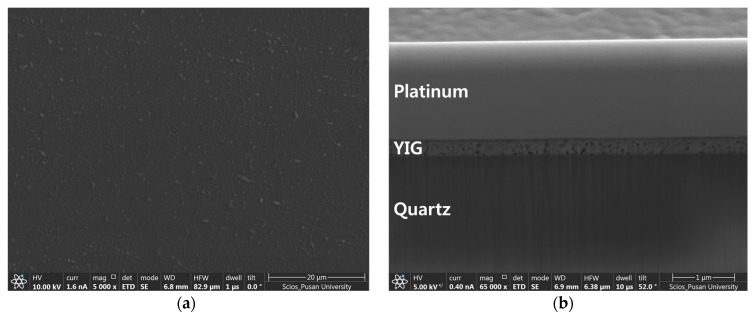
(**a**) A scanning electron microscopy image of the YIG sample (top-view); (**b**) A cross-section of the film. Note that the top platinum layer was deposited only for the ion milling by a focused ion beam, not for the thermoreflectance measurement.

The images of the sample surface and cross-section are presented in Figure 3. The film thickness is confirmed to be approximately 250 nm. The microstructural characteristics were discussed elsewhere [8]. The crystalline film is soft magnetic and has a highly homogeneous garnet structure without any side phases of YFeO_3_ or Fe_2_O_3_. Other physical and magnetic properties are summarized in Table 1.

**Figure 4:**
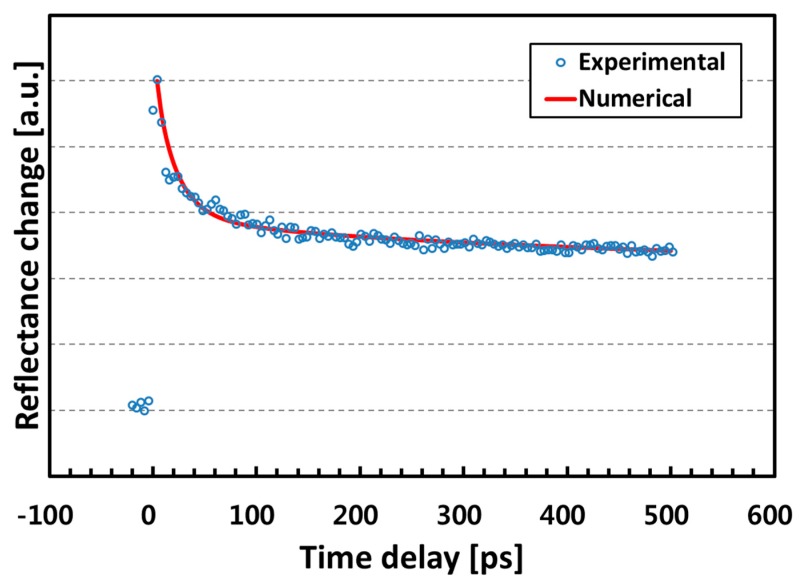
Thermoreflectance measurement data on the Al-coated quartz wafer. The numerical solution of the transient heat conduction equation (solid line) was curve fitted to the experimental data (open circle). A thermal conductivity value of 1.5 W/m·K was obtained for the quartz.

For calibration and validation purposes, the thermal conductivity of the quartz substrate was evaluated first. Figure 4 shows the numerical solution fitted to the thermoreflectance measurement data. Material properties used for the curve fitting are listed in Table 2. Thermal conductivities of 60 W/m·K and 1.5 W/m·K were obtained for Al film and quartz wafer, respectively, with a thermal boundary conductance of $\sigma_{K_{\mathrm{Al}/\mathrm{Quartz}}}$ = 1.1 × 10^8^ W/m^2^·K. The thermal conductivity of bulk pure Al typically lies in 204–273 W/m·K, but it is not surprising because the thermal conductivity could be smaller than the bulk value when it is in the form of a thin film due to the size effect [25,26,27,28]. Meanwhile, the thermal conductivity of quartz used in this work is close to the literature values (1.3–1.4 W/m·K).

Based on the measured thermal conductivity of Al, it is noticed that the optical energy of the laser does not heat up the entire film and substrate because the thermal diffusion length (*Λ*) is calculated as follows:

(15) $$\Lambda =\sqrt{\alpha t}$$

where *α* = *κ*/*ρC*_p_ is the thermal diffusivity. Using *κ* = 60 W/m·K and properties in Table 2, *t* = 198 ps is obtained for the film thickness of 70 nm, and this is sufficiently longer than the temporal pulse width of the laser beam. In addition, the temperature change in the film (Δ*T*) at the moment of pump-pulse application is determined by the following equation [29]:

(16) $$\Delta T(z)=(1-R)\frac{Q}{\rho C_{p}\xi A}\exp^{-z/\xi }$$

where *Q* is the pump-pulse energy delivered to the film surface, *ξ* = 1/*β* is the penetration depth, and *A* is the pump-spot area. The temperature rise at *z* = 0 is ~6 K for *Q* = 2 nJ and a pump-spot diameter of 50 μm.

**Figure 5:**
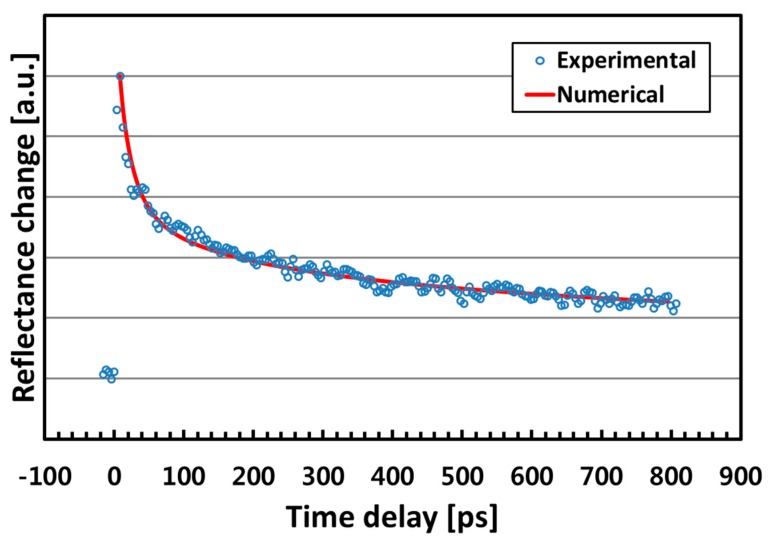
Thermoreflectance measurement data on the Al-coated YIG film. The curve fitting shows a thermal conductivity value of 11.0 W/m·K.

Figure 5 shows the evaluation result on the Al-coated YIG film. The thermoreflectance decay is faster than that of the quartz substrate, implying that the thermal conductivity of YIG is higher. The curve fitting was performed again and *κ*_YIG_ = 11.0 W/m·K gave good agreement. This can be converted to a thermal diffusivity value of 0.036 cm^2^/s using the density and specific heat values of 5170 kg/m^3^ [30] and 590 J/kg·K [4], respectively.

The thermal property is compared to literature values in Table 3. The film in the present study shows a higher thermal conductivity value than that of bulk YIG at room temperature, which is typically 7.4 W/m·K [31]. The result is also 22% higher than that of a 190 nm thick YIG film fabricated by the PLD technique [6]. Meanwhile, it is noted that the comparison may not necessarily show the thickness dependence of the thermal conductivity because films were made by different fabrication techniques that may result in different microstructures. If the thickness effect is to be clarified, films with varying thickness prepared by the same method have to be evaluated, ideally using a single measurement technique. In this sense, Table 3 rather provides only an overall idea on the variation of the thermal conductivity of YIG in different conditions. Nevertheless, it could still be an indication of the film quality since it influences the heat transport phenomena not only at cryogenic temperature but also at room temperature. For example, it was reported that the thermal conductivity increases as the average grain size increases due to the increase in the scattering rate of heat-carrying phonons by grain boundaries [32]. In addition, defects and impurities also promote the phonon-defect scattering process in YIG and consequently decrease the phonon mean free path [6,33,34]. Indeed, clarification of such effects would require rigorous and exhaustive studies on the microstructural characterization and magnetothermal measurements of nanoscale YIG thin films, not in the form of micrometer-thick or bulk materials, which is beyond the scope of this work and remains for future study.

## 5. Conclusions

The thermal conductivity of a nanoscale YIG film fabricated by a sol-gel method was evaluated in the present study. A 250 nm thick film with a single phase sample was prepared, and its thermal conductivity was measured using the ultrafast pump-probe technique. A numerical analysis was performed using the finite difference method to solve a transient heat conduction equation and to curve fit the solution to the experimental data. Results show that the thermal conductivity of as-fabricated film is 11.0 W/m·K, which is 22–83% higher than previously reported values obtained from different fabrication techniques. The thermal conductivity of YIG in this study implies the microstructural properties and film quality given by the sol-gel fabrication technique, considering the effects of defect concentration and film quality on the microscale heat transport.

## Figures and Tables

**Table 1:** Microstructural and magnetic properties of the yttrium iron garnet (YIG) sample [8].

Parameter	Value
Lattice constant (Å)	12.354
Crystal size (nm)	24
Saturation magnetization (emu/g)	6.1
Coercivity (Oe)	38

**Table 2:** Material properties used in the numerical analysis.

Material	Aluminum	YIG	Quartz
Density (kg/m^3^)	2700	5170	2203
Specific heat (J/kg·K)	900	590	740
Refractive index	2.23	-	-
Extinction coefficient	7.60	-	-
Optical reflectivity	0.88	-	-

**Table 3:** Thermal conductivity values from literature.

Reference	Sample Thickness (μm)	Deposition Technique	Characterization Technique	Measurement Temperature (K)	Thermal Conductivity (W/m·K)
Present study	0.250	Sol-gel	Ultrafast pump-probe	300	11.0
[6]	0.190	Pulsed laser deposition	3*ω* method	275	9.0
[4]	1.0 × 10^3^	Liquid phase epitaxy	Laser flash method	300	8.0
[17]	2.5 × 10^3^	Traveling solvent floating zone	Steady-state method	300	8.8
[33]	11.3 × 10^3^	PbO–PbF_2_ flux	Steady-state method	300	7.4
[35]	12.7 × 10^3^	Sintering	Laser flash method	296	6.0
